# Supplementary figures and images for: Necroptosis increases with age in the brain and contributes to age-related neuroinflammation
Source: GeroScience. 2021 Sep 13;43(5):2345–61. doi: 10.1007/s11357-021-00448-5 (PMC8599532; doi:10.1007/s11357-021-00448-5)

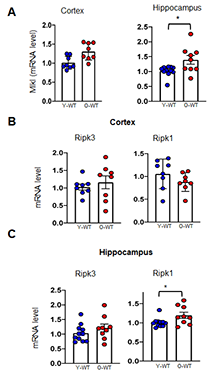

Supplement: Supplementary file 1 — Transcript levels of MLKL in the cortex (left panel) and hippocampus (right panel) of young WT (Y-WT, red) and old-WT (O-WT, blue) mice (A). Transcript levels of RIPK3 and RIPK1 in the cortex (B) and hippocampus (C) of Y-WT and O-WT mice. Data were obtained from 8 to 10 mice per group and are expressed as the mean ± SEM. * p ≤ 0.05). (PNG 35 kb) [file 11357_2021_448_Fig6_ESM.png]

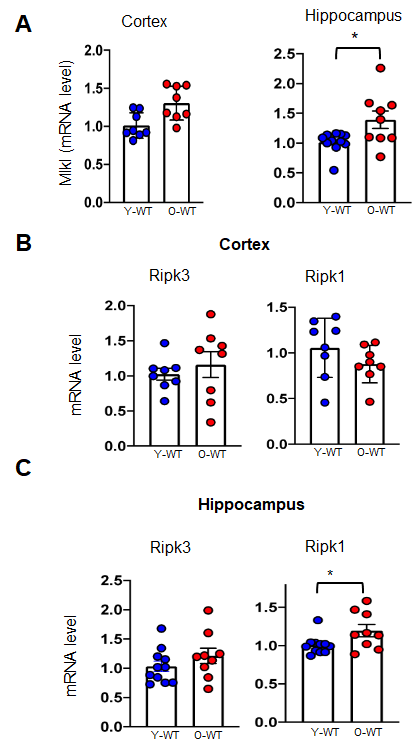

Supplement: Supplementary file 2 — High Resolution Image (TIFF 1101 kb) [file 11357_2021_448_MOESM1_ESM.tiff]

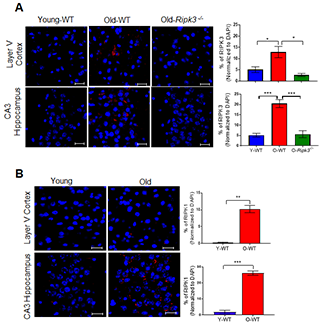

Supplement: Supplementary file 3 — Confocal images showing expression of RIPK3 (A) and RIPK1 (B) in the layer V of the cortex and the CA3 region of the hippocampus of young WT (Y-WT), old WT (O-WT), and old Ripk3-/- mice. RIPK3 and RIPK1 staining is in red and DAPI staining is in blue (left panel). Graphical representation of the percentage of RIPK3 or RIPK1 normalized to DAPI for Y-WT (blue bar), O-WT (red bar), and old Mlkl-/- mice (green bar) are shown in the right panel. Data were obtained from 5-7 mice per group and are expressed as the mean ± SEM. * p ≤ 0.05, **p≤ 0.01, ***p≤ 0.001. Scale bar: 20 μm. (PNG 58 kb) [file 11357_2021_448_Fig7_ESM.png]

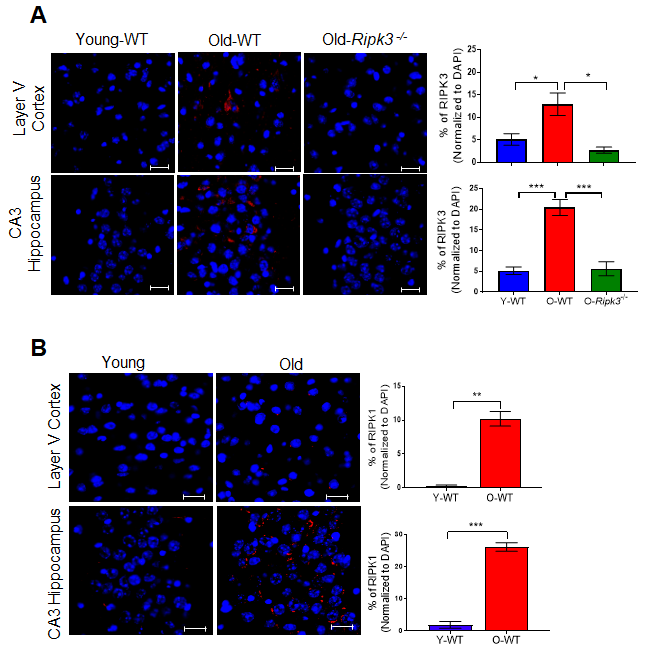

Supplement: Supplementary file 4 — High Resolution Image (TIFF 1466 kb) [file 11357_2021_448_MOESM2_ESM.tiff]

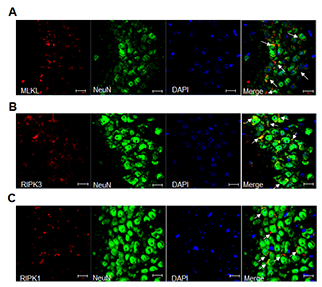

Supplement: Supplementary file 5 — Confocal images showing expression of MLKL (A), RIPK3 (B) and RIPK1(C) in neurons at the CA3 region of the hippocampus. Double immunofluorescence staining and confocal micrographs are shown for neurons (NeuN, green), MLKL or RIPK3 or RIPK1 (red) and DAPI (blue). White arrows indicate the co-localization of MLKL or RIPK3 or RIPK1 with NeuN. Scale bar: 20 μm. (PNG 65 kb) [file 11357_2021_448_Fig8_ESM.png]

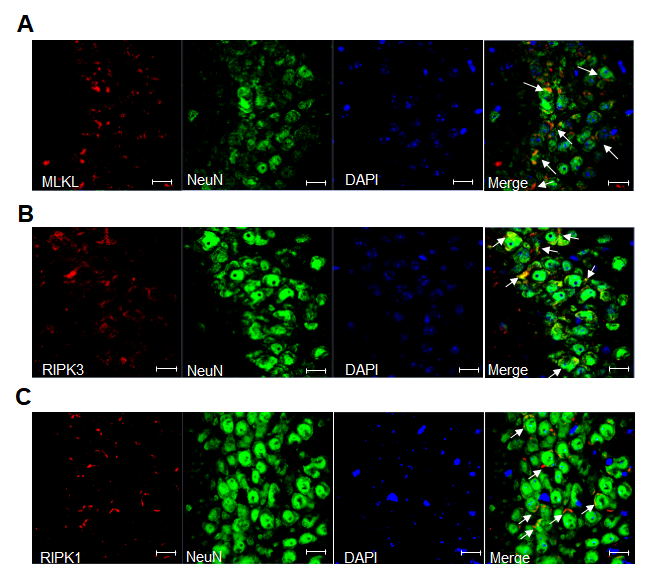

Supplement: Supplementary file 6 — High Resolution Image (TIFF 1359 kb) [file 11357_2021_448_MOESM3_ESM.tiff]
